# Profound Neutropenia with Concomitant Hepatitis During Oxacillin Therapy

**DOI:** 10.7759/cureus.3823

**Published:** 2019-01-03

**Authors:** John B Miller, Allan C Gelber

**Affiliations:** 1 Rheumatology, Johns Hopkins University School of Medicine, Baltimore, USA

**Keywords:** beta-lactam, adverse drug reactions, osteomyelitis, oxacillin

## Abstract

Beta-lactam antibiotics are widely used and generally well-tolerated. Neutropenia and hepatitis are two adverse drug reactions associated with beta-lactam therapy that are typically benign and reversible, yet have seldom been reported as simultaneous occurrences. This narrative describes these adverse drug reactions during oxacillin therapy.

## Introduction

Oxacillin is commonly used for the management of methicillin-susceptible *Staphylococcus aureus* infections. While oxacillin and its broader class of beta-lactam antibiotics are generally well-tolerated, oxacillin can induce diarrhea, rash, and nephritis. Cytopenias and hepatotoxicity have also been described with the use of beta-lactam antimicrobial therapy, though simultaneous occurrence is uncommon [1-2].

## Case presentation

A 19-year-old man with a history of a left lower extremity gunshot wound requiring a popliteal-tibial bypass first presented with pain over the dorsum of his left great toe. An overlying area with ulceration was probed to the bone. He was febrile to 38.3^0^C and tachycardic at 110 bpm with leukocytosis of 14,800 WBC/mm^3^ and was commenced empirically on vancomycin and piperacillin-tazobactam therapy. Deep wound cultures grew methicillin-sensitive *Staphylococcus aureus*, and his antibiotic regimen was narrowed to oxacillin, 2 grams every four hours. Given his prior intravenous drug use, he was discharged to a nursing facility to complete a six-week course of intravenous antibiotic therapy for osteomyelitis.

He was readmitted four weeks later after a behavioral disturbance led to premature discharge from the facility. Admission laboratory data demonstrated profound neutropenia (30/mm^3^) and a marked elevation in liver transaminases (aspartate aminotransferase (AST) 339 U/L, alanine aminotransferase (ALT) 551 U/L) (Table 1). The differential diagnosis of acute hepatic injury with pronounced neutropenia included antibiotic toxicity, and oxacillin was promptly discontinued. Cefazolin was administered for the final two weeks of his antibiotic course. The neutropenia resolved within days while hepatitis resolved over the subsequent two weeks (Table 1).

**Table 1:** Lab Results AST: aspartate aminotransferase; ALT: alanine aminostransferase; WBC: white blood cell count; ANC: absolute neutrophil count

	Range	Two years prior	Admission labs from first hospitalization	One week after oxacillin	Four weeks after oxacillin	One week off oxacillin	Conclusion of antibiotic therapy
AST	0-37 U/L	20	71	17	339	123	24
ALT	0-40 U/L	20	82	39	551	114	21
Alkaline phosphatase	30-120 U/L	72	169	139	206	143	102
Total bilirubin	0.1-1.2 mg/dL	0.3	0.5	0.1	0.2	0.1	0.1
WBC	4,500-13,000/cu mm	8,000	14,800	9,900	1,800	6,600	6,200
ANC	1,500-7,800/cu mm	---	13,000	---	30	3,500	4,000

## Discussion

Concomitant neutropenia and hepatitis due to beta-lactam therapy are exceedingly rare and seldom reported [1-2]. In a prospective study of 128 consecutive patients receiving high-dose cloxacillin (150 mg/kg/d), 17% (n=22) developed neutropenia (<2 x106/L) and 5% (n=7) developed hepatitis (i.e., AST or ALT >45 IU/L) [3]. Notably, only one patient developed both neutropenia and hepatitis. Neutropenia was associated with a longer duration and higher cumulative doses of antibiotic therapy and was more common among those <30 years of age. In another prospective study of 41 patients receiving oxacillin, 37% (n=15) developed hepatitis (i.e., an increase in transaminase level from normal to >100 IU/L); only 12% (n=5) were directly attributed to oxacillin [4]. All were male and between 21 and 37 years of age; each was switched to cefazolin with an improvement in liver enzymes, typically occurring four to 16 days after beta-lactam discontinuation. Leukocyte counts remained uniformly normal, even at maximal elevation in transaminase values.

The mechanism of neutropenia is incompletely understood. Bone marrow aspirates from patients with suspected oxacillin-induced neutropenia identify hypercellular matrix with the arrest of the myeloid lineage, disproportionately affecting granulopoiesis [5]. Such cytopenia seemingly occurs with higher doses and extended oxacillin courses, though it remains unclear if drug-induced neutropenia is due to direct bone marrow toxicity or an immune-mediated injury. Many patients with oxacillin-induced neutropenia also develop fever, rash, and eosinophilia, suggestive of a hypersensitivity reaction [2,6]. Interestingly, the addition of various beta-lactam agents to bone marrow cultures demonstrates a direct, dose-dependent inhibition of myeloid colony formation, suggesting a direct role in marrow toxicity [7]. This would also explain the rapid granulocyte and leukocyte count recovery typically observed after drug discontinuation.

Oxacillin-induced hepatotoxicity can present as a hepatocellular injury or cholestasis. Whereas typical neutrophil recovery can be robust within a few days, recovery from hepatitis is often delayed. The etiology of liver injury from oxacillin is also incompletely understood. Flucloxacillin-induced hepatotoxicity has previously been associated with HLA-B*5701, suggesting an immune-mediated process, though the isoxazole ring on oxacillin is also thought to contribute directly to liver injury [2,8].

Our patient was treated with a standard adult dose of oxacillin for osteomyelitis. However, given his weight of 60 kilograms, this equated to 200 mg/kg/day, well above the recommended pediatric dose of 100 mg/kg/day. The Food and Drug Administration (FDA) recommends monitoring cell counts on a weekly basis and a periodic assessment of liver function while on oxacillin. As above, staphylococcal skin and bone infections are common disorders in the medicine service. This instructive case of a dose-related adverse drug reaction may raise awareness among treating physicians to the potentially harmful effect of beta-lactam antibiotics to the bone marrow, liver, or concomitantly to both organ systems, particularly among thin young adults.

## Conclusions

This narrative underscores the potentially harmful effect of beta-lactam antibiotics to bone marrow and liver, particularly among thin young adults. In this case of concomitant oxacillin-induced neutropenia with hepatitis, medication withdrawal resulted in rapid laboratory improvement.
